# Identification of novel *RIPK4* variants in a Chinese patient with Arthrogryposis Multiplex Congenita (AMC)

**DOI:** 10.1186/s13052-025-01858-3

**Published:** 2025-01-21

**Authors:** Yi-Lei Lu, Meng-wei Liu, Jie-Yuan Jin, Ding Pan

**Affiliations:** 1 Department of Orthopaedics, Xiangya Hospital of Central South University, Changsha, China; 2 Microsurgery & Reconstruction Research Center, Xiangya Hospital of Central South University, Changsha, China; 3 School of Life Sciences, Central South University, Changsha, China; 4 College of Basic Medical, Xinjiang Medical University, Urumqi, China

**Keywords:** Receptor-Interacting Protein Kinase 4, Arthrogryposis multiplex congenital, Whole-exome sequencing, Sanger sequencing

## Abstract

**Background:**

Arthrogryposis multiplex congenita (AMC) is a congenital disorder characterized by multiple joint involvement, primarily affecting limb mobility and leading to various tissue contractures. Variations in the *RIPK4* gene may impact connective tissues, thereby resulting in a spectrum of malformations. This study aimed to identify the genetic etiologies of AMC patients and provide genetic testing information for further diagnosis and treatment of AMC.

**Methods:**

We recruited a Chinese female patient with hand-related AMC and her family members. Whole-exome sequencing (WES) was employed to determine the genetic etiologies of the patient’s disease. The pathogenic mechanisms of the identified variations were analyzed using protein tolerance profiling and modeling.

**Results:**

We identified two novel *RIPK4* variants (c.1354G > A, p.E452K; c.1558A > T, p.T520S). Pathogenicity studies indicated that the c.1354G > A, p.E452K variant changed the charge from negative to positive and altered the chemical properties from acidic to alkaline, potentially significantly affecting protein function.

**Conclusions:**

We reported the discovery of two novel *RIPK4* variants (c.1354G > A, p.E452K; c.1558A > T, p.T520S) in a Chinese AMC female patient’s family. Our study enhances the genetic repository for AMC and highlights the pathogenicity of *RIPK4* variants, underscoring the significance of comprehensive management for genetic-related diseases, particularly the critical roles of prenatal diagnosis and genetic counseling.

**Trial registration:**

The research protocol received approval from the Ethics Review Committee of Xiangya Hospital of Central South University in China (approval number: 202103427), registered in March 2021, with all participants providing duly signed informed consent forms.

**Supplementary Information:**

The online version contains supplementary material available at 10.1186/s13052-025-01858-3.

## Introduction

Arthrogryposis Multiplex Congenita (AMC) is a congenital disorder characterized by the involvement of multiple joints across different parts of the body, predominantly affecting the upper and lower limbs. Clinically, it manifests as a loss of joint mobility, restricted extension, or increased stiffness, often accompanied by varying degrees of deformity, pain, dislocation, and other complications [1]. Significantly, the disease exhibits pronounced heterogeneity, presenting a complex clinical profile with contractured tissues that include the skin, neurovascular structures, joint capsules, ligaments, tendons, muscles, and cartilage [2].

The incidence of AMC is estimated to be about 1 in every 3000 to 5000 live births [3]. Its etiology is multifaceted, encompassing a combination of maternal environmental factors and fetal genetic elements. These factors collectively inhibit fetal intrauterine activity, thereby inducing congenital multiple joint contractures [4]. Abnormal uterine shapes, multiple pregnancies, specific viral infections during pregnancy [5] (including Newcastle virus, Akabane virus, and Coxsackie virus), as well as the presence of multiple sclerosis [6, 7], are considered factors that restrict fetal movement within the womb, consequently increasing the risk of AMC.

Moreover, genetic factors play a significant role in the pathogenesis of arthrogryposis. The use of whole-genome sequencing in recent years has significantly advanced the identification of numerous genes associated with AMC. Gina Ravenscroft et al. [8] have demonstrated that 42% of cases of fetal akinesia and AMC received accurate genetic diagnoses through whole-exome sequencing, identifying genes such as *CACNA1S*, *CHRNB1*, *GMPPB*, and *STAC3* and describing phenotypic expansions associated with these genes. In a comprehensive study, Annie Laquerriere et al. [2] discovered nine newly identified genes: *CNTNAP1*, *MAGEL2*, *ADGRG6*, *ADCY6*, *GLDN*, *LGI4*, *LMOD3*, *UNC50*, and *SCN1A*, and also identified pathogenic variants in *ASXL3* and *STAC3*, expanding the phenotypes associated with these genes.

It is noteworthy that variants in more than 220 genes have been associated with arthrogryposis [9]. However, the molecular etiology remains unknown for a large number of cases. Nonetheless, the potential of whole-exome studies in revealing the intricate nature of AMC is undeniable. For instance, exome sequencing studies have revealed key findings, such as *MYH3* gene mutations leading to abnormal encoding of skeletal muscle contractile proteins and *ECEL1* gene mutations interfering with the normal formation of neuromuscular junctions, both of which are associated with severe AMC [9]. Therefore, this study is committed to identifying new genetic variants in a family with AMC, contributing to the growing body of knowledge regarding the genetic underpinnings and potential pathways of AMC. This research expands the genetic mutation library and may deepen our understanding of its heterogeneity and complexity, also aims to provide patients with more precise prognostic assessments and accurate genetic counseling, while laying the groundwork for the development of more precise diagnostic methods and innovative treatment strategies.

## Materials and methods

### Subjects

The research protocol received approval from the Ethics Review Committee of Xiangya Hospital of Central South University in China (approval number: 202103427), with all participants providing duly signed informed consent forms. The focus of this study was a Chinese family with a history of Arthrogryposis Multiplex Congenita (AMC). The proband (II:1) sought medical consultation at the Orthopedics Department of Xiangya Hospital in 2021, accompanied by her parents (I:1 and I:2). Comprehensive data encompassing family medical history, physical examination records, and blood samples of the subjects were meticulously collected for subsequent analysis.

### DNA extraction

Blood samples were treated with an anticoagulant containing EDTA to prevent coagulation. DNA was extracted from the peripheral blood lymphocytes of the patient and the patient’s parents using the using the DNeasy Blood and Tissue Kit (Qiagen, Valencia, California).

### Whole exome sequencing and Sanger sequencing

**Fig. 1:**
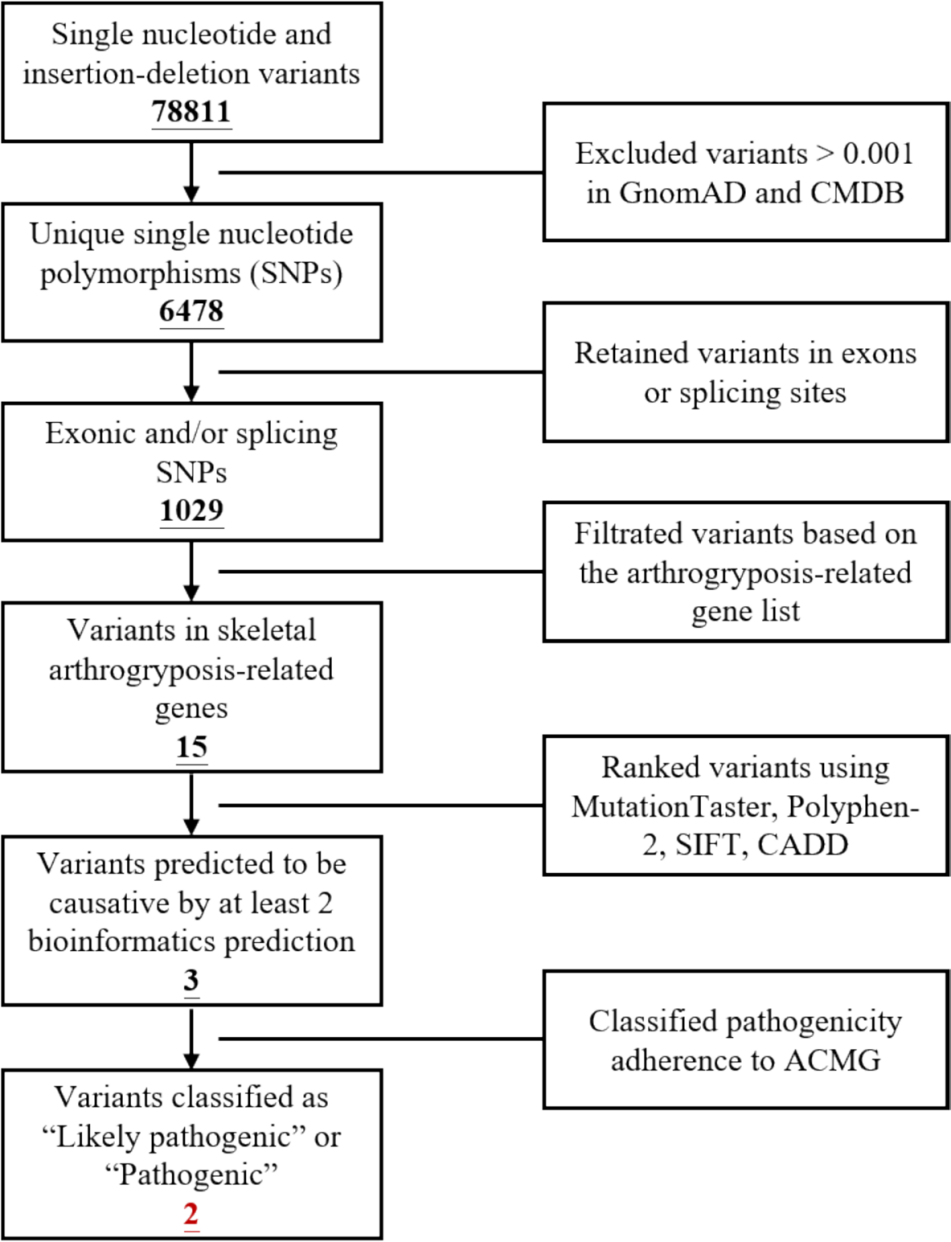
Workflow diagram depicting the processes of WES and Sanger sequencing. WES, whole-exome sequencing

The arthrogryposis-related genes are summarized in Table S1, and we adopted the WES technology to screen these variants listed in the table. The main part of WES was provided by the Berry Genomics Company Limited (Chengdu, China), as previously described [10]. All the exomes were captured by Agilent Sure Select Human All Exon V6 kits and sequenced by Illumina HiSeq X Ten platform. Conservation analysis of the amino acids at the detected mutation sites was conducted using Mutation Taster (https://www.mutationtaster.org/). The detailed procedures for Whole Exome Sequencing and Sanger Sequencing are illustrated in Fig. 1. In this study, we employed several computational tools to predict the potential impact of genetic variants on protein function. Specifically, we utilized Polyphen-2 (http://genetics.bwh.harvard.edu/pph2/), SIFT (http://provean.jcvi.org/index.php), MutationTaster (https://www.mutationtaster.org/), and CADD (https://cadd.gs.washington.edu/snv/). To determine the frequencies of these variants, we consulted data from the GnomAD database (http://gnomad.broadinstitule.org) and the Chinese Millionome Database (CMDB; http://cmdb.bgi.com/cmdb/), excluding variants with a minor allele frequency (MAF) greater than 0.001 in both databases. Our approach to annotating candidate genes with respect to their inheritance patterns and clinical phenotypes strictly adhered to the guidelines established by the Online Mendelian Inheritance in Man (OMIM) database (https://www.omim.org). The classification of variant pathogenicity was conducted in accordance with the standards and guidelines set forth by the American College of Medical Genetics and Genomics (ACMG) [11].

The primer pairs employed for PCR amplification were meticulously designed using Primer Premier 5.0 software, and the nucleotide sequences can be provided upon request (Forward Primer: 5’- CACAGACGTCCAGAAGAAGAAG-3’, Reverse Primer: 5’- CTTGGCGTTGACACTGATCT-3’; Forward Primer: 5’-GCTGCTCAACAATGCCAAC-3’, Reverse Primer: 5’-CAGGATGCGCACGATATTCT-3’).

### Variants protein tolerance profiling and modeling

The landscape of tolerance to amino acid substitutions in the RIPK4 protein was generated using the MetaDome web tool (https://stuart.radboudumc.nl/metadome/dashboard). *RIPK4* coding sequence was analyzed for tolerance to variation, and the visualization was created based on a combination of multiple sequence alignment and population genetic data. A color-coded representation was utilized, with blue indicating regions of the protein that are tolerant to substitutions and red indicating regions where substitutions are likely deleterious. P.E452K and p.T520S, were annotated on the landscape to investigate their potential impact on protein function. The full-length human RIPK4 protein structure (amino acids 784) was predicted using AlphaFold2. The forecasted conformation was visualized and analyzed for interactions using ChimeraX. Layout and annotation of the predicted structure were executed with Adobe Illustrator for presentation purposes.

## Results

### Case description

**Fig. 2:**
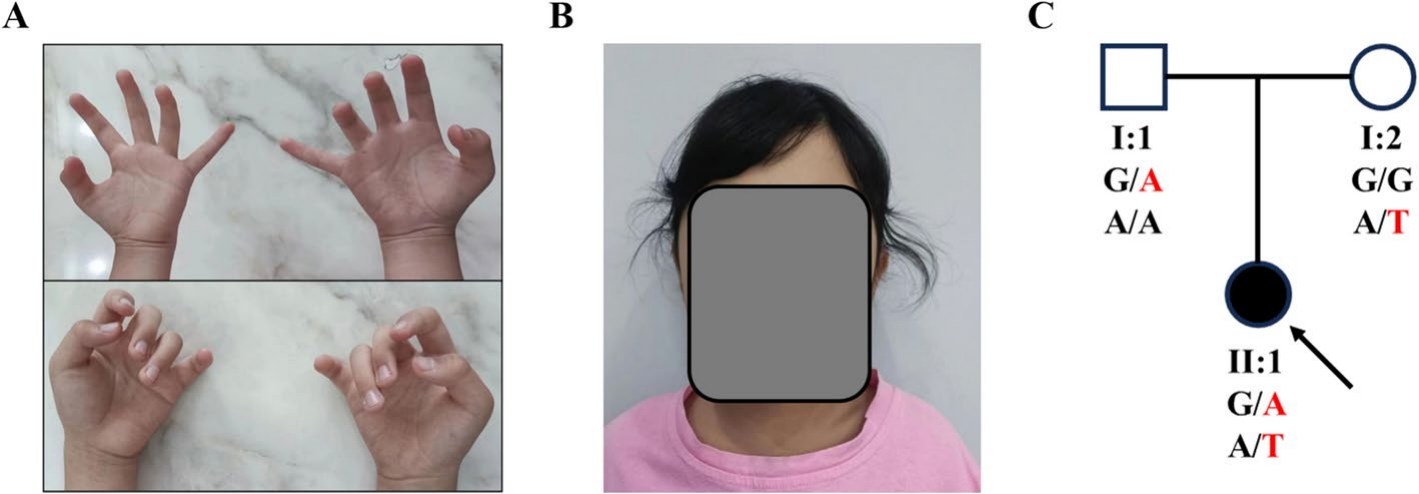
**A** Clinical assessment of the patient’s hands showing restricted mobility. **B** The patient’s phenotype presenting curly hair. **C** Family history chart. Both parents are carriers of an autosomal recessive variant, and the patient is a homozygous individual for this condition

A 9-year-old female presented at the Orthopedic Outpatient Clinic of Xiangya Hospital, Central South University, with a history of restricted hand movements since infancy (Fig. 2A). Physical examination revealed symmetrical contractures of the fingers joints in both hands. The fingers appeared curved, with the thumbs flexed and adducted across the palms. There was slight muscular atrophy in the hands, and the metacarpophalangeal, interphalangeal, and distal interphalangeal joints were rigid in a flexed position. Both active and passive flexion and extension were limited, and passive movements elicited pain. The skin over the joints showed diminished normal texture, appeared mildly shiny, and was taut; these alterations impeded daily activities such as grasping and writing, although the sensory function of the hand skin remained intact. Multidisciplinary consultation revealed that the patient had curly hair (Fig. 2B), normal growth and development, no narrow forehead or microcephaly, normal neurodevelopment, no strabismus or ptosis, and good bilateral visual acuity, without scapular winging. The first child of healthy, non-consanguineous parents, she was born at term via a natural delivery without prenatal diagnosis. Joint contractures were noted several months postnatally, leading to a diagnosis of congenital multiple joint contractures. Notably, there was no similar medical history in the family, making her the first in the family to exhibit these symptoms (Fig. 2C). Following consultation and in alignment with patient preferences, comprehensive exome sequencing was recommended to identify the etiology and assess genetic likelihood, accompanied by a six-month regimen of orthotic traction therapy and regular follow-up evaluations.

### Genetic analysis

**Fig. 3:**
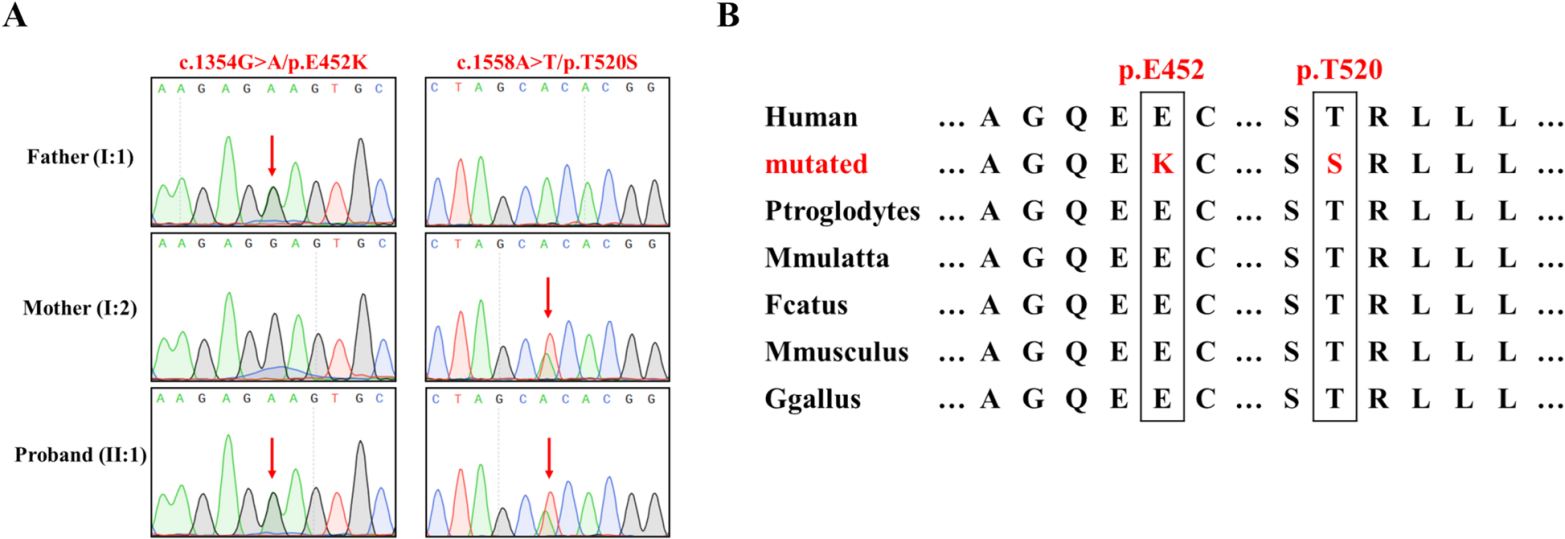
**A** Sanger sequencing results for the family’s *RIPK4* gene. The panel shows the presence of a significant variant in the father’s *RIPK4* gene (c.1354G > A:p.E452K) and a distinct variant in the mother’s *RIPK4* gene at a different locus (c.1558A > T:p.T520S). These findings confirm that the identified variants in the proband follow an autosomal recessive inheritance pattern, leading to pathogenic symptoms when both variants are present. **B** Conservation analysis of the *RIPK4* variants. The image demonstrates the high degree of conservation for both variants (c.1354G > A:p.E452K and c.1558A > T:p.T520S) across different species, as indicated by Mutation Taster analysis

Whole Exome Sequencing (WES) generated a substantial 13.44 Gb of data, achieving an extensive 99.9% coverage of the designated target region, and 98.6% of this target region was covered at a depth exceeding 10 × . Subsequent genetic analysis, which focused on genes implicated in arthrogryposis (Table S1), was employed to refine the selection from the 15 remaining variants identified in the proband, leading to the identification of a set of 3 variants across 3 different genes. Notably, two novel variants (c.1354G > A, p.E452K; c.1558A > T, p.T520S) in the *RIPK4* gene, associated with Arthrogryposis Multiplex Congenita (AMC), were unearthed (Table 1). Sanger sequencing was used to confirm the presence of the *RIPK4* variant in the proband and to determine the genotypes of other family members, revealing a significant variant in the father’s *RIPK4* gene (c.1354G > A:p.E452K) and a different variant in the mother’s *RIPK4* gene at another locus (c.1558A > T:p.T520S) (Fig. 3A). These findings indicate that the proband inherited one variant from each parent, leading to pathogenic symptoms in an autosomal recessive manner when both variants are present. Mutation taster analysis indicated that both variants are highly conserved (Fig. 3B).

**Table 1:** Variant identified in the patient with AMC by WES

Gene	OMIM clinical phenotype	Variant	Allele Frequency	Pathogenicity prediction	American College of Medical Genetics classification
*RIPK4*	AR: CHAND syndrome, Popliteal pterygium syndrome, Bartsocas-Papas type 1	NM_020639.3:exon8:c.1354G > A:p.E452K	Unknown variant	MutationTaster: D SIFT: D Polyphen-2: D	Likely pathogenic (PM1, PM2, PP1, PP3)
		NM_020639.3:exon8:c.1558A > T:p.T520S	Unknown variant	MutationTaster: D SIFT: D Polyphen-2: D	Likely pathogenic (PM1, PM2, PP1, PP3)

*D* disease causing, *AR* Autosomal recessive

**Fig. 4:**
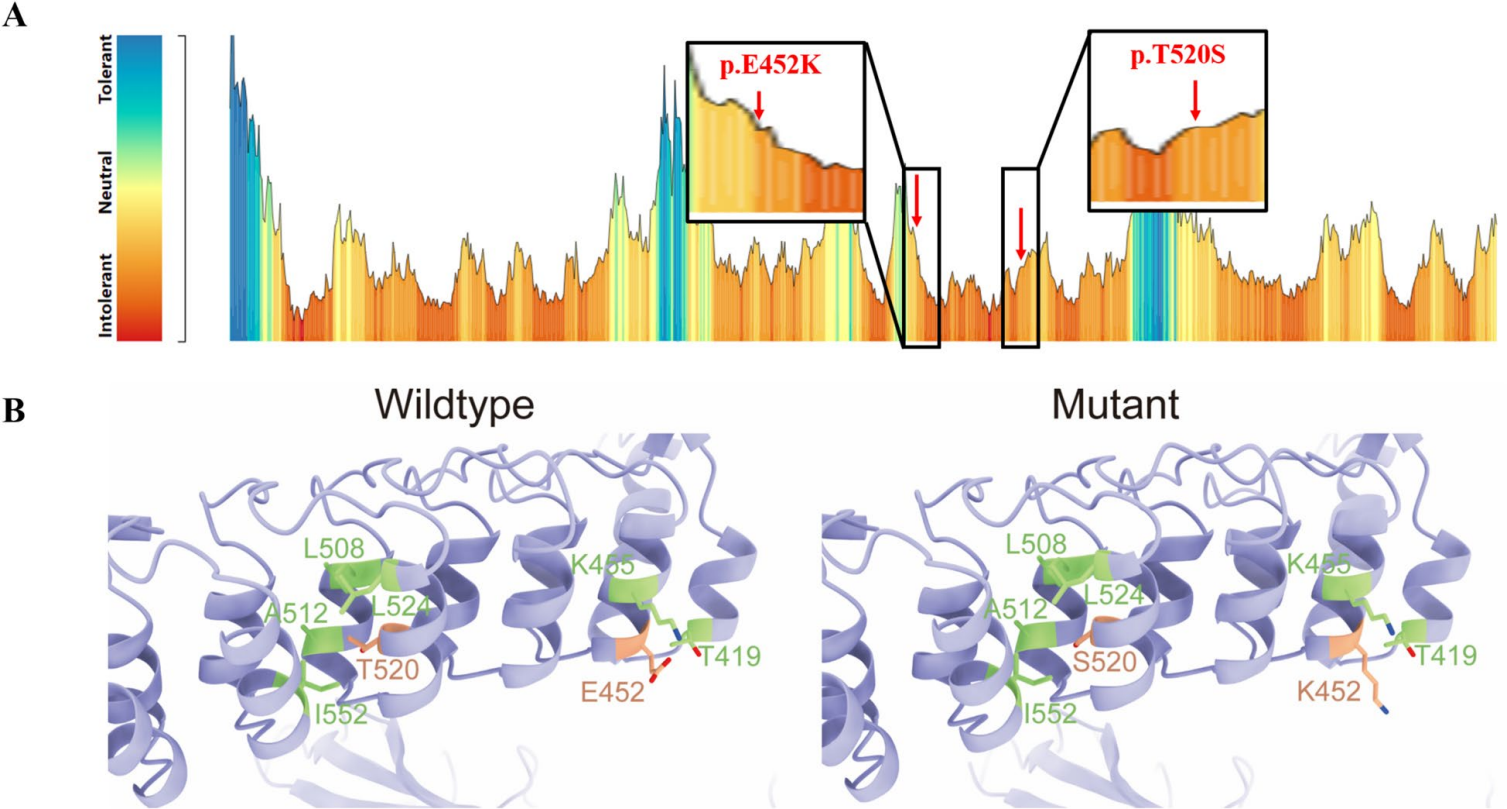
**A** Tolerance landscape of RIPK4 protein variants. The color-coded gradient represents the degree of predicted tolerance to amino acid substitutions along the *RIPK4* protein sequence, with blue indicating high tolerance and red indicating low tolerance. Notable substitutions, p.E452K and p.T520S, are highlighted, demonstrating regions of potential functional impact. The gradient scale on the left denotes the transition from tolerant (top) to intolerant (bottom) regions. **B** Comparison of protein structures at the mutation site between the wild type and mutant type

The MetaDome analysis of the RIPK4 protein revealed a variable tolerance landscape for amino acid substitutions along its sequence (Fig. 4A). The majority of the protein sequence exhibited regions that are neutral to tolerant towards substitutions. However, several peaks of intolerance (red) indicate regions that are likely critical for the protein’s function, as they are less tolerant to variation. Notably, the mutation p.E452K is located in a moderately intolerant region, suggesting a potential deleterious effect on RIPK4 function. Contrarily, the p.T520S mutation is found in a highly intolerant region, indicating a significant functional impact and a strong likelihood of being deleterious.

As shown in Fig. 4B, in the wild-type human RIPK4 protein, the threonine at position 520 (T520) engaged in hydrophobic interactions with the neighboring residues L508, A512, L524, and I552. The substitution of this residue to serine (S) as a variant did not substantially alter the pre-existing hydrophobic environment. However, the side chain of glutamic acid at position 452 (E452) was oriented towards the exterior of the protein, not engaging in interactions with adjacent amino acids such as T419 and K455. Nonetheless, the variant resulting in lysine (K) at this position converted the charge from negative to positive, thereby affecting the original hydrophilic environment. Thus, we considered the two *RIPK4* variants (c.1354G > A:p.E452K; c.1558A > T:p.T520S) to be likely pathogenic for the proband.

## Discussion

In our research, we utilized Whole Exome Sequencing (WES) and Sanger sequencing to investigate potential genetic abnormalities in a patient diagnosed with Arthrogryposis Multiplex Congenita (AMC). This examination led to the identification of two novel variants in the *RIPK4* gene, designated as c.1354G > A:p.E452K and c.1558A > T:p.T520S. Notably, these newly discovered *RIPK4* variants (c.1354G > A:p.E452K; c.1558A > T:p.T520S) were not present in the Genome Aggregation Database (GnomAD) or the China Metabolic Disease Database (CMDB).

In accordance with the American College of Medical Genetics and Genomics (ACMG) guidelines, we determined the pathogenicity of the *RIPK4* variants as “likely pathogenic.” This classification was based on two moderate pieces of evidence (PM1 and PM2) and two supporting pieces of evidence (PP1 and PP3), as outlined in Table 1: (1) Both *RIPK4* variants were located in regions known for frequent mutations (PM1); (2)these variants were absent in control datasets from GnomAD and CMDB (PM2); (3)these variants co-segregated with the disease in family studies (PP1); (4)Multiple prediction software tools, including MutationTaster, SIFT, and Polyphen-2, indicated that these variants were likely to have deleterious impacts on the gene or its products (PP3).

Following our tolerance profiling (Fig. 3) and modeling analyses (Fig. 4) of the two *RIPK4* variants (c.1354G > A:p.E452K and c.1558A > T:p.T520S), we considered them likely to be pathogenic. The T520S variant has a minor impact on the protein’s hydrophobic environment, whereas the E452K variant, due to the change in charge, alters the chemical nature of the site from acidic to basic, potentially exerting a more significant effect on protein function. Further experimental or clinical correlation analyses are needed in the future to ascertain the pathogenicity of these variants in relation to Arthrogryposis Multiplex Congenita (AMC).

The proband’s parents each carried a single variant in the *RIPK4* gene, exhibiting no pathological phenotypes. Indeed, these variants were inherited by the proband in an autosomal recessive manner, leading to symptoms when both variants are present in the homozygous state. Colin Campbel et al. [12] highlights that pathogenic variants often occur in evolutionarily conserved areas. While a single variant might not impact these areas significantly, multiple variants can lead to notable functional changes and disease​​. Yuki Saito’s [13] research supports this by suggesting that the combination of multiple variants can increase disease penetrance​​. Similarly, Hong Sun’s [14] study on predicting pathogenicity of single or multiple nsSNPs in genes like *WFS1* shows how interactions between these variants can exacerbate diseases​​. These findings collectively suggest that while individual variants in *RIPK4* may not cause significant protein dysfunction or disease, their combination can be pathogenic due to cumulative effects in key conserved regions.

Table 2 summarizes the pathogenic and likely pathogenic mutations of the *RIPK4* gene that have been reported in recent years, primarily associated with Bartsocas-Papas syndrome and CHANDS (Curly Hair-Ankyloblepharon-Nail Dysplasia Syndrome). Bartsocas-Papas syndrome is characterized by developmental anomalies such as cleft palate, limb deformities, and skin defects, while CHANDS features include abnormal hair, eyelid fusion (ankyloblepharon), and nail dysplasia. Kalay et al. [15] reported a major pathogenic mutation in the *RIPK4* gene that leads to Bartsocas-Papas syndrome: c.362 T > A, resulting in the amino acid at position 121 changing from isoleucine to asparagine (p.Ile121Asn), indicating an impact on RIPK4’s stability and kinase activity, and leading to severe multiple malformations. Similarly, in a Kuwaiti family also reported to have Bartsocas-Papas syndrome, Gollasch et al. [16] identified a *RIPK4* mutation: c.850G > A, causing the amino acid at position 284 to change from glutamic acid to lysine (p.Glu284Lys). This mutation, located outside the kinase domain, differs from typical mutation sites in Bartsocas-Papas syndrome, suggesting that different locations of *RIPK4* mutations may lead to varying degrees of phenotype severity. De Groote et al. [17] discussed the mechanism of action of RIPK4, emphasizing its interaction with IRF6 in regulating epidermal fusion, a common feature in Bartsocas-Papas syndrome, suggesting a crucial role through a common molecular pathway in epidermal fusion. The consistency of these findings confirms the critical role of the *RIPK4* gene in skin and organ formation.

**Table 2:** List of pathogenic variants reported in *RIPK4*

Number	Mutation		Clinical Phenotype	Classification
1	c.121del	p. H41fs	not provided	Likely pathogenic
2	c.242 T > A	p. I81N	Bartsocas-Papas syndrome 1dup	Pathogenic
3	c.362 T > A	p. I121N	Bartsocas-Papas syndrome 1dup	Pathogenic
4	c.722G > A	p. R241H	Bartsocas-Papas syndrome 1dup	Likely pathogenic
5	c.777dup	p. R260fs	Bartsocas-Papas syndrome 1dup	Pathogenic
6	c.850G > A	p. E284K	Curly hair, ankyloblepharon, nail dysplasia syndrome	Pathogenic
7	c.1074dup	p. E359*	Bartsocas-Papas syndrome 1dup	Pathogenic
8	c.1127C > A	p. S376*	Bartsocas-Papas syndrome 1dup	Pathogenic

The * in the genetic notation indicates that the position is replaced by a stop codon

The Receptor-Interacting Protein Kinase 4 (RIPK4) plays a crucial role in various biological processes, particularly in epidermal development and maintenance. It is a member of the RIPK family, initially identified as an interacting partner of protein kinase C (PKC) β and PKCδ. RIPK4 is known to regulate several signaling pathways, including NF-κB, Wnt/β-catenin, and RAF/MEK/ERK ones, which are vital for keratinocyte differentiation, cutaneous inflammation, and wound repair [18]. The mutations in *RIPK4* might disrupt these critical signaling pathways, potentially affecting keratinocyte differentiation and skin development. Since skin and connective tissues play a role in joint mobility and integrity, disturbances in these pathways could feasibly contribute to the development of joint contractures seen in AMC. However, the exact mechanisms by which *RIPK4* mutations might lead to AMC are not fully elucidated and would require further investigation.

Besides investigating pathogenic mechanisms, the prevention and diagnosis of genetic-related diseases should also be emphasized. This concept of full-course management not only improves the current condition of patients but also reduces the overall incidence of genetic diseases through prevention and early diagnosis. Prenatal diagnostic techniques, such as non-invasive prenatal testing (NIPT), amniocentesis, and chorionic villus sampling, can detect if a fetus carries specific genetic disorders early [19]. This is a crucial decision-making tool for prospective parents, aiding them in making informed pregnancy choices. Genetic counseling plays a key role in this process, helping families understand the risks of genetic diseases and assess risks based on their genetic background and family history [20]. The phenotypic diversity of genetic diseases presents diagnostic challenges, but prenatal genetic testing plays a crucial role in early diagnosis, helping parents better understand their fetus’ health [21]. Additionally, high-throughput sequencing technologies, such as whole-genome sequencing, can detect large-scale structural variations in the genome, including deletions, duplications, and other types of copy number variations; whole-exome sequencing makes it possible to identify specific gene mutations within a relatively short time [22]. The application of these technologies greatly facilitates the diagnosis of rare genetic diseases and the discovery of new pathogenic genes [19, 23]. The patient currently shows hand contracture and curly hair, with no disorders in the eyes, growth, or nervous system, partially consistent with previous studies. In addition to the current treatment plans for hand contracture, multidisciplinary regular follow-ups should be conducted [24], focusing on monitoring the child’s growth and development, and the nervous and respiratory systems to identify potential symptoms early [25]. Although the proband’s parents did not undergo prenatal testing, we have identified the child’s genotype through whole-exome sequencing and can offer genetic counseling and prenatal diagnostic recommendations in the future.

## Conclusion

Our study reported a case of Arthrogryposis Multiplex Congenita (AMC) and identified two novel *RIPK4* variants (c.1354G > A:p.E452K and c.1558A > T:p.T520S) through Whole Exome Sequencing (WES) and Sanger sequencing. Bioinformatics analysis of the patient’s phenotypic and functional changes indicated the pathogenic nature of these *RIPK4* variants and their association with AMC. Our findings contribute to the expanding spectrum of *RIPK4* variants, uncovering the pathogenicity related to alterations in the acidity and basicity at specific sites within RIPK4 that affect protein structural stability. This research further strengthens the correlation between the phenotypic expression and genetic mutations of AMC, laying a solid foundation for future exploration of the consequences associated with AMC-related variants. In light of these genetic insights, we particularly emphasized the importance of comprehensive management of genetic-related diseases, where prenatal diagnosis and genetic counseling play a crucial role.

## Supplementary Information

Supplementary Material 1.

## Data Availability

Data sharing is not applicable to this article as the data involves patient privacy. Please contact the corresponding author for data.

## References

[1] Ma L, Yu X. Arthrogryposis multiplex congenita: classification, diagnosis, perioperative care, and anesthesia. Front Med. 2017;11(1):48–52. 10.1007/s11684-017-0500-4. 28213879 10.1007/s11684-017-0500-4

[2] Laquerriere A, Jaber D, Abiusi E, et al. Phenotypic spectrum and genomics of undiagnosed arthrogryposis multiplex congenita. J Med Genet. 2022;59(6):559–67. 10.1136/jmedgenet-2020-107595. 33820833 10.1136/jmedgenet-2020-107595 PMC9132874

[3] Lowry RB, Sibbald B, Bedard T, Hall JG. Prevalence of multiple congenital contractures including arthrogryposis multiplex congenita in Alberta, Canada, and a strategy for classification and coding. Birth Defects Res A Clin Mol Teratol. 2010;88(12):1057–61. 10.1002/bdra.20738. 21157886 10.1002/bdra.20738

[4] Rink BD. Arthrogryposis: a review and approach to prenatal diagnosis. Obstet Gynecol Surv. 2011;66(6):369–77. 10.1097/OGX.0b013e31822bf5bb. 21851751 10.1097/OGX.0b013e31822bf5bb

[5] Kirkland PD, Barry RD, Harper PA, Zelski RZ. The development of Akabane virus-induced congenital abnormalities in cattle. Vet Rec. 1988;122(24):582–6. 10.1136/vr.122.24.582. 3137718 10.1136/vr.122.24.582

[6] Livingstone IR, Sack GH. Arthrogryposis multiplex congenita occurring with maternal multiple sclerosis. Arch Neurol. 1984;41(11):1216–7. 10.1001/archneur.1984.04050220118031. 6487109 10.1001/archneur.1984.04050220118031

[7] Riemersma S, Vincent A, Beeson D, et al. Association of arthrogryposis multiplex congenita with maternal antibodies inhibiting fetal acetylcholine receptor function. J Clin Invest. 1996;98(10):2358–63. 10.1172/JCI119048. 8941654 10.1172/JCI119048 PMC507687

[8] Ravenscroft G, Clayton JS, Faiz F, et al. Neurogenetic fetal akinesia and arthrogryposis: genetics, expanding genotype-phenotypes and functional genomics. J Med Genet. 2021;58(9):609–18. 10.1136/jmedgenet-2020-106901. 33060286 10.1136/jmedgenet-2020-106901 PMC8328565

[9] Bayram Y, Karaca E, CobanAkdemir Z, et al. Molecular etiology of arthrogryposis in multiple families of mostly Turkish origin. J Clin Invest. 2016;126(2):762–78. 10.1172/JCI84457. 26752647 10.1172/JCI84457 PMC4731160

[10] Jin JY, Wu PF, Luo FM, et al. GLIS Family Zinc Finger 1 was First Linked With Preaxial Polydactyly I in Humans by Stepwise Genetic Analysis. Front Cell Dev Biol. 2022;9:781388. 10.3389/fcell.2021.781388. 35087831 10.3389/fcell.2021.781388 PMC8787328

[11] Richards S, Aziz N, Bale S, et al. Standards and Guidelines for the Interpretation of Sequence Variants: A Joint Consensus Recommendation of the American College of Medical Genetics and Genomics and the Association for Molecular Pathology. Genet Med. 2015;17(5):405–24. 10.1038/gim.2015.30. 25741868 10.1038/gim.2015.30 PMC4544753

[12] Campbell C, Francis A, Gaunt TR. Predicting pathogenicity from non-coding mutations. Nat Biomed Eng. 2023;7(6):709–10. 10.1038/s41551-022-00996-x. 36543871 10.1038/s41551-022-00996-x

[13] Saito Y, Koya J, Araki M, et al. Landscape and function of multiple mutations within individual oncogenes. Nature. 2020;582(7810):95–9. 10.1038/s41586-020-2175-2. 32494066 10.1038/s41586-020-2175-2

[14] Sun H, Yu G. New insights into the pathogenicity of non-synonymous variants through multi-level analysis. Sci Rep. 2019;9(1):1667. 10.1038/s41598-018-38189-9. 30733553 10.1038/s41598-018-38189-9 PMC6367327

[15] Kalay E, Sezgin O, Chellappa V, et al. Mutations in RIPK4 cause the autosomal-recessive form of popliteal pterygium syndrome. Am J Hum Genet. 2012;90(1):76–85. 10.1016/j.ajhg.2011.11.014. 22197489 10.1016/j.ajhg.2011.11.014 PMC3257895

[16] Gollasch B, Basmanav FB, Nanda A, et al. Identification of a novel mutation in RIPK4 in a kindred with phenotypic features of Bartsocas-Papas and CHAND syndromes. Am J Med Genet A. 2015;167A(11):2555–62. 10.1002/ajmg.a.37233. 26129644 10.1002/ajmg.a.37233

[17] De Groote P, Tran HT, Fransen M, et al. A novel RIPK4-IRF6 connection is required to prevent epithelial fusions characteristic for popliteal pterygium syndromes. Cell Death Differ. 2015;22(6):1012–24. 10.1038/cdd.2014.191. 25430793 10.1038/cdd.2014.191 PMC4423184

[18] Xu J, Wei Q, He Z. Insight Into the Function of RIPK4 in Keratinocyte Differentiation and Carcinogenesis. Front Oncol. 2020;10:1562. 10.3389/fonc.2020.01562. 32923402 10.3389/fonc.2020.01562 PMC7457045

[19] Serra G, Corsello G, Antona V, et al. Autosomal recessive polycystic kidney disease: case report of a newborn with rare PKHD1 mutation, rapid renal enlargement and early fatal outcome. Ital J Pediatr. 2020;46(1):154. 10.1186/s13052-020-00922-4. 33059727 10.1186/s13052-020-00922-4 PMC7560064

[20] Serra G, Felice S, Antona V, et al. Cardio-facio-cutaneous syndrome and gastrointestinal defects: report on a newborn with 19p13.3 deletion including the MAP 2 K2 gene. Ital J Pediatr. 2022;48(1):65. 10.1186/s13052-022-01241-6. 35509048 10.1186/s13052-022-01241-6 PMC9069788

[21] Piro E, Serra G, Giuffrè M, Schierz IAM, Corsello G. 2q13 microdeletion syndrome: Report on a newborn with additional features expanding the phenotype. Clinical Case Reports. 2021;9(6):e04289. 10.1002/ccr3.4289.

[22] Serra G, Antona V, Giuffrè M, et al. Interstitial deletions of chromosome 1p: novel 1p31.3p22.2 microdeletion in a newborn with craniosynostosis, coloboma and cleft palate, and review of the genomic and phenotypic profiles. Ital J Pediatr. 2022;48(1):38. 10.1186/s13052-022-01232-7. 35246213 10.1186/s13052-022-01232-7 PMC8896361

[23] Piccione M, Serra G, Sanfilippo C, Andreucci E, Sani I, Corsello G. A new mutation in EDA gene in X-linked hypohidrotic ectodermal dysplasia associated with keratoconus. Minerva Pediatr. 2012;64(1):59–64. 22350046

[24] Serra G, Giambrone C, Antona V, et al. Congenital hypopituitarism and multiple midline defects in a newborn with non-familial Cat Eye syndrome. Ital J Pediatr. 2022;48(1):170. 10.1186/s13052-022-01365-9. 36076277 10.1186/s13052-022-01365-9 PMC9461219

[25] Schierz IAM, Serra G, Antona V, Persico I, Corsello G, Piro E. Infant developmental profile of Crisponi syndrome due to compound heterozygosity for CRLF1 deletion. Clin Dysmorphol. 2020;29(3):141–3. 10.1097/MCD.0000000000000325. 32433043 10.1097/MCD.0000000000000325

