# Supplementary material for: Identification of novel RIPK4 variants in a Chinese patient with Arthrogryposis Multiplex Congenita (AMC)
Source: Ital J Pediatr. 2025 Jan 21;51:6. doi: 10.1186/s13052-025-01858-3 (PMC11748807; doi:10.1186/s13052-025-01858-3)
Supplement: Supplementary file 1 — Supplementary Material 1. [file 13052_2025_1858_MOESM1_ESM.docx]

**Supplementary Table 1.** The arthrogryposis-related genes list.

| ACTA1 | ANTXR2 | CANT1 | CHRNB1 | CNTNAP1 | COL6A2 | DHCR7 | ECEL1 | ERCC6 |
| --- | --- | --- | --- | --- | --- | --- | --- | --- |
| FGF9 | FKTN | GLE1 | HOXD13 | KIAA0196 | MASP1 | MUSK | NSD1 | PEX14 |
| PFKM | PMP22 | PRX | RAPSN | RNASEH2C | SHOX | SOX9 | TNNT3 | UBA1 |
| ZC4H2 | ADAMS10 | AP1S2 | CASK | CHRND | COFS | COL6A3 | DNM2 | EIF2S3 |
| ERG2 | FGFR1 | FLNA | GLI3 | HSPG2 | KIF7 | MED12 | MYBPC1 | OFD1 |
| PEX2 | PIEZO2 | PMPK | PTDSS1 | RBM10 | RYR1 | SLC05A1 | SULF1 | TPM2 |
| UBE1 | ZIC3 | ADAMTSL2 | ARX | CATSAL | CHRNE | COL1A1 | COL7A1 | DOK7 |
| EMG1 | ESCO2 | FGFR2 | FLNB | GLRA1 | IMPAD1 | LARGE | MHS3 | MYH3 |
| PAFAH1B1 | PEX26 | PIP5KIC | POMGNT1 | PTHR | RELN | SAMHD1 | SLC26A2 | SYNE1 |
| TPM3 | UPK3A | ADCY6 | ASXL1 | CATSHL | CHRNG | COL1A2 | COLEC10 | DRG2 |
| ERBB3 | FBN1 | FGFR3 | FLVCR2 | GLRB | INSR | LICAM | MMP2 | MYH8 |
| PAX3 | PEX3 | PLOD1 | POMT1 | PTLAH | RET | SCARF2 | SLC9A6 | TARP |
| TREX1 | VIPAR | ADGRG6 | ATRX | CD96 | CHST14 | COL1A5 | COLEC11 | DTDST |
| ERCC1 | FBN2 | FHL1 | GBA | GPC3 | IRF6 | LIS1 | MNX1 | NEB |
| PEHO | PEX5 | PLOD2 | POMT2 | RAB18 | RIPK4 | SELENON | SLCO5A1 | TBX22 |
| TRPV4 | VPS33B | ADSL | B3GAT3 | CHAT | CMG2 | COL2A1 | CRLF1 | DYM |
| ERCC2 | FCMD | FKBP10 | GDF5 | GRUL3 | KAT6B | LMBR1 | MPZ | NF1 |
| PEX1 | PEX6 | PM11 | POR | RAB3GAP1 | RNASEH2A | SEPN2 | SMARCAD1 | TBX5 |
| TSC1 | WNT7A | AKT1 | BIN1 | CHRNA1 | CNTN1 | COL6A1 | DCX | EBP |
| ERCC5 | FGD1 | FKRP | GJA1 | HOXA13 | KCNA1 | LMX1B | MTM1 | NOG |
| PEX12 | PEX7 | PMM2 | PRG4 | RAB3GAP2 | RNASEH2B | SETBP1 | SMN | TNNI2 |
| TSC2 | ZBTB42 |  |  |  |  |  |  |  |
